# Supplementary material for: Quasi-equilibrium phase coexistence in single component supercritical fluids
Source: Nat Commun. 2021 Jul 30;12:4630. doi: 10.1038/s41467-021-24895-y (PMC8324840; doi:10.1038/s41467-021-24895-y)
Supplement: Supplementary file 1 — Supplementary Information [file 41467_2021_24895_MOESM1_ESM.pdf]

SUPPLEMENTARY INFORMATION

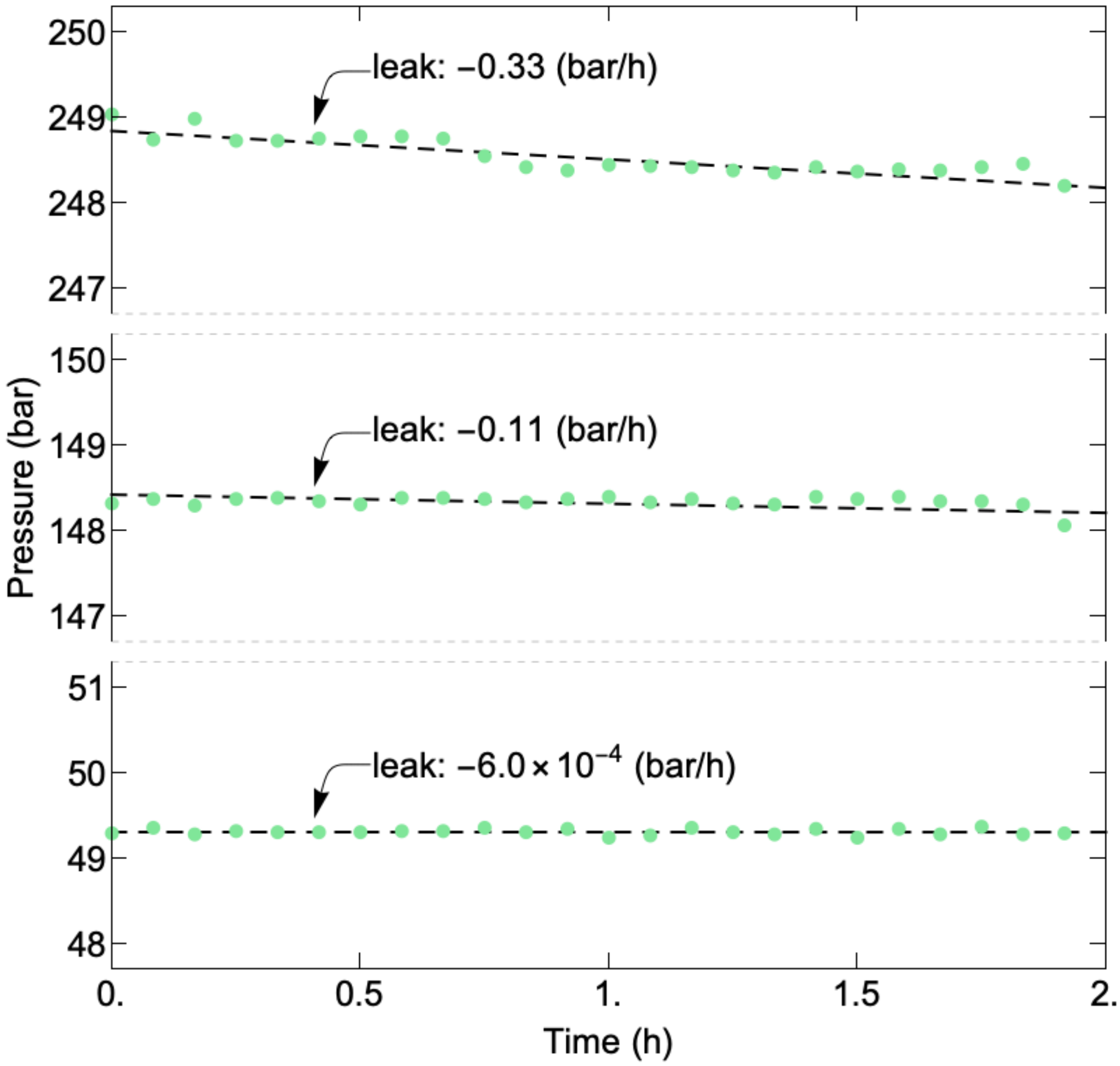

Supplementary Fig.1 | The high pressure chamber leak test results. The chamber securely preserves the internal pressure for the experimental timescale.
