# Supplementary material for: Quasi-equilibrium phase coexistence in single component supercritical fluids
Source: Nat Commun. 2021 Jul 30;12:4630. doi: 10.1038/s41467-021-24895-y (PMC8324840; doi:10.1038/s41467-021-24895-y)
Supplement: Supplementary file 3 — Description of Additional Supplementary Files [file 41467_2021_24895_MOESM3_ESM.pdf]

Description of Additional Supplementary Files

File name: Supplementary Movie 1
Description: The Brownian motion of droplets in the highpressure chamber. The chamber is filled with argon to 100 bar. The droplets are individually identifiable due to their strong scattering of the laser light compared to the background, which makes trackable of their Brownian motions. The mean size of droplets is estimated by statistical analysis of the Brownian motions.
